# Validation of reference genes from Eucalyptus spp. under different stress conditions

**DOI:** 10.1186/1756-0500-5-634

**Published:** 2012-11-14

**Authors:** Jullyana Cristina Magalhães Silva Moura, Pedro Araújo, Michael dos Santos Brito, Uiara Romero Souza, Julianade Oliveira Fernandes Viana, Paulo Mazzafera

**Affiliations:** 1 Departamento de Ciências Biológicas, Instituto de Ciências Exatas, Naturais e Educação, Universidade Federal do Triângulo Mineiro, Av. Getúlio Guaritá 159, Bairro Abadia, Uberaba, MG, 38025-440, Brazil; 2 Departamento de Biologia Vegetal, Instituto de Biologia, CP 6109, Universidade Estadual de Campinas, Campinas, SP, 13083-970, Brazil; 3 Centro Avançado da Pesquisa Tecnológica do Agronegócio de Cana, Ribeirão Preto, SP, CP 206 14001-970, Brazil; 4 Fibria Celulose S.A., Rodovia Euryale de Jesus Zerbine, Km 84, São Silvestre, Jacareí, SP, 12340-010, Brazil

**Keywords:** *Eucalyptus*, Drought, Cold, Light, Reference genes

## Abstract

**Background:**

The genus *Eucalyptus* consists of approximately 600 species and subspecies and has a physiological plasticity that allows some species to propagate in different regions of the world. Eucalyptus is a major source of cellulose for paper manufacturing, and its cultivation is limited by weather conditions, particularly water stress and low temperatures. Gene expression studies using quantitative reverse transcription polymerase chain reaction (qPCR) require reference genes, which must have stable expression to facilitate the comparison of the results from analyses using different species, tissues, and treatments. Such studies have been limited in eucalyptus.

**Results:**

*Eucalyptus globulus* Labill, *Eucalyptus urograndis* (hybrid from *Eucalyptus urophylla* S.T. Blake X *Eucalyptus grandis* Hill ex-Maiden) and *E. uroglobulus* (hybrid from *E. urograndis* X *E. globulus*) were subjected to different treatments, including water deficiency and stress recovery, low temperatures, presence or absence of light, and their respective controls. Except for treatment with light, which examined the seedling hypocotyl or apical portion of the stem, the expression analyses were conducted in the apical and basal parts of the stem. To select the best pair of genes, the bioinformatics tools *GeNorm* and *NormFinder* were compared. Comprehensive analyses that did not differentiate between species, treatments, or tissue types, showed that *IDH* (isocitrate dehydrogenase), *SAND* (SAND protein), *ACT* (actin), and *A-Tub* (α-tubulin) genes were the most stable. *IDH* was the most stable gene in all of the treatments.

**Conclusion:**

Comparing these results with those of other studies on eucalyptus, we concluded that five genes are stable in different species and experimental conditions: *IDH*, *SAND*, *ACT*, *A-Tub*, and *UBQ* (ubiquitin). It is usually recommended a minimum of two reference genes is expression analysis; therefore, we propose that *IDH* and two others genes among the five identified genes in this study should be used as reference genes for a wide range of conditions in eucalyptus.

## Background

The genus *Eucalyptus* belongs to the Myrtaceae family, which consists of approximately 600 species and subspecies, exhibits physiological plasticity with worldwide dispersion and successfully grows in different regions. Eucalyptus is a major source of cellulose for manufacturing paper [1] and globally, Brazil ranks first in the production of short fibre cellulose [2]. Due to its economic importance, the genome sequencing of the species *Eucalyptus grandis* was recently completed [3].

*E. globulus* and *E. grandis* are the two main species cultivated in the world [4]. Among the existing species, *E. globulus* is known for its resistance to cold temperatures and its lower content and type (greater amount of S units) of lignin, which results in a higher extraction yield of cellulose [5-7]. *E. globulus* also exhibits tolerance to cold weather, which allows it to be cultivated in areas where other species do not grow well. In Brazil, the hybrid *E. urograndis* (hybrid of *E. urophylla* x *E. grandis*) has been widely cultivated for its unique features, compared with other cultivated species [8,9]. It has been reported that more than 600,000 acres are cultivated with this hybrid species in Brazil, being the basis of the Brazilian silviculture for the production of cellulose and paper [10]. *E. uroglobulus* is a hybrid derived from *E. urograndis x E. globulus*, with a high cellulose yield [11].

One of the most widely used approaches for molecular characterisation is gene expression analysis, which aims to identify genes that can be used as molecular markers or for genetic manipulations. The most common technique for analysing gene expression is quantitative reverse transcription polymerase chain reaction (qPCR), which is advantageous because of its speed, sensitivity and specificity [12].

One of the most important aspects in gene expression analysis by qPCR is the determination of genes that have constitutive expression, which will serve as references in the analysis of plant material from different origins. Thus, the expression of these genes must be constant, regardless of the tissue under analysis comes from different parts of the same plant or plants from different origins. Such genes are called normalisers because it is possible to compare gene expression in various types of biological materials [13-15] based on the individual expression of these genes. However, genes that have previously been described as adequate normalisers for some plant species and/or experimental conditions are not always valid for other species or working conditions [16]. Typically, the selected endogenous genes are related to cellular maintenance processes that are common among different cell types [17]. The use of the appropriate normaliser genes is a limitation to the use of qPCR, and this justifies the considerable number of published studies aimed at identifying useful endogenous genes for the study of gene expression in different species, including rice [18], sugarcane [19], *A. thaliana*[20], potato [21], *Brachypodium* spp. [22], tomato [23], coffee [24], wheat [25], tobacco [26] and *Eucalyptus* spp. [27-30].

de Oliveira [27] performed an extensive search of 21,432 eucalyptus genes in *E. grandis* leaves and xylem and *E. globulus* xylem. The genes were evaluated using microarray hybridisation analysis, and 50 candidate genes were identified that showed little variation in their expression. These genes were evaluated in gene expression studies associated with the flower, leaf, and xylem of six species of eucalyptus. de Almeida et al. [30] aimed to identify endogenous genes for gene expression studies associated with adventitious rooting in *E. globulus* microcuttings because low rooting efficiency is a problem for the cloning and multiplication of this species. Fernández et al. [28] attempted to identify normaliser genes in *E. globulus* for gene expression studies related to cold acclimation. Similarly, Boava et al. [29] sought to identify normaliser genes for studies of Urograndis hybrid resistance to leaf rust caused by *Puccinia psidii*.

None of these studies identified normaliser genes in eucalyptus that could be widely used in gene expression studies related to cold and drought stress in species of significant economic importance, such as *E. urograndis*, *E. uroglobulus*, and *E. globulus*. Low temperature and limited water are stresses that limit the growth of eucalyptus [11,31-35]. In this study, we evaluated normaliser genes in *E. urograndis*, *E. uroglobulus*, and *E. globulus* plants exposed to low temperature and stressed with water shortage. The recovery of the water stressed plants after re-watering was also evaluated. Additionally, we also evaluated normaliser genes in the seedlings and plants of *E. globulus* grown in the presence and absence of light, which have been used in lignification studies in this species (Araújo and Mazzafera, unpublished). The validation of endogenous genes under different growth conditions could enable their use in a broad range of studies of gene expression in eucalyptus. In our evaluations we compared the bioinformatics programs *NormFinder* and *GeNorm*.

## Results

Several comparisons were performed combining species, treatments, tissues and all data using both *GeNorm* and *NormFinder*. The results of these analyses are shown in Table 1, which was split into four sections to improve the presentation of the data.

**Table 1:** Identification of the best pairs of genes for different treatments, species, and tissues

**A**							
**Species**	**Software**	**CTRL**	**DRT**	**REC**	**CTRL+DRT+REC Apex**	**CTRL+DRT+REC Base**	**All treatments**
***E. globulus***	GeNorm	A-Tub/UBQ	A-Tub/H2B	IDH/SAND	UBQ/H2B	H2B/A-Tub	H2B/UBQ
	NormFinder	IDH/ACT	ACT/A-Tub	A-Tub/IDH	IDH/A-Tub	A-Tub/ACT	A-Tub/ACT
***E. urograndis***	GeNorm	SAND/A-Tub	H2B/UBQ	A-Tub/SAND	UBQ/A-Tub	IDH/ACT	IDH/ACT
	NormFinder	ACT/SAND	UBQ/H2B	SAND/A-Tub	ACT/SAND	A-Tub/SAND	ACT/SAND
***E. uroglobulus***	GeNorm	H2B/UBQ	EF1/IDH	IDH/ACT	SAND/IDH	IDH/18S	A-Tub/ACT
	NormFinder	A-Tub/EF1	SAND/ACT	18S/H2B	H2B/A-Tub	A-Tub/ACT	A-Tub/H2B
**All species**	GeNorm	UBQ/H2B	IDH/SAND	IDH/ACT	IDH/ACT	IDH/ACT	IDH/ACT
	NormFinder	IDH/SAND	ACT/IDH	SAND/IDH	SAND/ACT	SAND/IDH	SAND/IDH
**B**							
**Species**	**Software**	**CTRL**	**COLD**	**CTRL+COLD Apex**	**CTRL+COLD Base**	**All treatments**	
***E. globulus***	GeNorm	A-Tub/UBQ	EF1/IDH	EF1/IDH	SAND/EF1	EF1/IDH	
	NormFinder	ACT/UBQ	SAND/UBQ	ACT/A-Tub	ACT/SAND	ACT/A-Tub	
***E. urograndis***	GeNorm	IDH/UBQ	A-Tub/UBQ	UBQ/A-Tub	UBQ/18S	A-Tub/UBQ	
	NormFinder	IDH/UBQ	SAND/UBQ	SAND/IDH	ACT/IDH	IDH/SAND	
***E. uroglobulus***	GeNorm	IDH/ACT	UBQ/SAND	SAND/UBQ	B-tub/SAND	UBQ/SAND	
	NormFinder	A-Tub/H2B	ACT/IDH	H2B/UBQ	A-Tub/ACT	H2B/A-Tub	
**All species**	GeNorm	SAND/UBQ	ACT/SAND	UBQ/IDH	IDH/SAND	IDH/SAND	
	NormFinder	A-Tub/EF1	IDH/ACT	H2B/UBQ	IDH/SAND	IDH/SAND	
**C**							
**Species**	**Software**	**DRT+COLD Apex**	**DRT+COLD Base**	**All treatments**			
***E. globulus***	GeNorm	IDH/A-Tub	IDH/SAND	IDH/ACT			
	NormFinder	UBQ/IDH	UBQ/A-Tub	UBQ/A-Tub			
***E. urograndis***	GeNorm	H2B/UBQ	SAND/A-Tub	IDH/ACT			
	NormFinder	ACT/A-Tub	SAND/A-Tub	ACT/IDH			
***E. uroglobulus***	GeNorm	IDH/ACT	EF1/IDH	IDH/ACT			
	NormFinder	H2B/A-Tub	A-Tub/ACT	A-Tub/H2B			
**All species**	GeNorm	ACT/IDH	IDH/SAND	IDH/ACT			
	NormFinder	ACT/IDH	SAND/IDH	IDH/ACT			
**D**							
**Species**	**Software**	***in vitro *(dark and light)**	**Greenhouse (dark and night)**	**All treatments**			
***E. globulus***	GeNorm	H2B/IDH	EF1/ACT	UBQ/IDH			
	NormFinder	UBQ/ACT	IDH/EF1	ACT/IDH			

A – Water stress experiment; B – Low-temperature experiment; C – Water stress + low-temperature experiments in tissues (apex and base), D – light experiments. CTRL = control; DRT – water stress; REC – recovery from water stress; COLD – low temperature.

### GeNorm

In the water stress trial, the analyses using *GeNorm* showed a wide range of genes, depending on the combination of treatments, species, and types of tissues analysed (Table 1A). For *E. globulus,* the most stable pair of genes, regardless of treatment or stem position, was *H2B*/*UBQ*. *IDH*/*ACT* and *A-Tub*/*ACT* were the best pairs for *E. urograndis* and *E. uroglobus*, respectively. When analysing the best pair of genes among all of the situations (treatments, species, and types of tissues), the most stable pair was *IDH*/*ACT*. Among the nine genes analysed, only five were observed in the combinations using GeNorm: *IDH*, *UBQ*, *ACT*, *A-Tub* and *H2B*.

In the low-temperature experiment, a large variation between the gene pairs was observed, depending on the type of treatment, species and tissue combination (Table 1B). In the general analyses within each species *IDH*, *UBQ*, *A-Tub*, *SAND* and *EF1* emerged as the most stable. From the general analysis, disregarding treatments, species, and types of tissues, the best gene pair was *IDH/SAND*.

Using *GeNorm*, the comparison of tissue type (Table 1C) without distinction between treatments, tissue types and species showed the gene pair *IDH*/*ACT* as the most stable. *GeNorm* indicated that *UBQ*/*IDH* was the best gene pair from the analyses with seedlings and plants of *E. globulus* with or without exposure to light (Table 1D).

### NormFinder

When *NormFinder* was used, several pairs of genes were identified and like in the *GeNorm* analysis, they varied depending on the treatment, species, and plant tissue. In the water stress treatments (Table 1A), at least one of the genes identified was consistently different from those suggested using *GeNorm*. For example, in the evaluation of *E. urograndis*, the general analysis of the treatments and tissues resulted in the identification of *IDH*/*ACT* by *GeNorm* and *ACT*/*SAND* by *NormFinder*. There were cases that both genes of the pair were different, such as for *E. globulus,* where *H2B*/*UBQ* and *A-Tub*/*ACT* were identified by *GeNorm* and *NormFinder*, respectively. In the analysis grouping the data from the water stress experiments, which included all species, treatments (control, water stress and recovery) and tissue types (apex and base), the observed most stable gene pair was *SAND*/*IDH*. When the low-temperature data were analysed altogether (Table 1B), the gene pair *IDH*/*SAND* was identified as the best combination for *NormFinder*. Among the nine genes analysed, five genes were present among all of the possible pairs during the water stress and low-temperature treatments when the analysis was carried out with *NormFinder*: *A-Tub*, *ACT*, *SAND*, *H2B* and *IDH*.

When comparing the tissue types (Table 1C), the best combination of primers for all species was *IDH*/*ACT*, which was also the same pair of genes identified in the analysis of plants/seedlings of *E. globulus* subjected to light and dark treatments (Table 1D).

**Figure 1:**
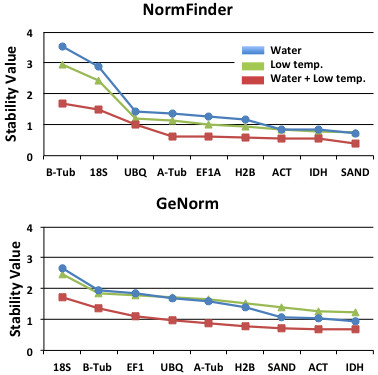
**The stability values of the genes calculated according to the two software programmes used in the analyses of the most stable genes.** The graph shows the analyses differentiating the water stress treatments (control + water stress + water stress recovery), the temperature treatments (control + low temperature), and both treatments together, regardless of species or tissue types (apex and base).

In the general analysis, i.e., all treatments, species and tissues types, *SAND*, *IDH* and *ACT* presented the lower stability values, indicating they were the most stable genes (Figure 1).

## Discussion

In this study, we used three *Eucalyptus* species in experiments where plants were exposed to different water regimes (sufficient, stressed, and recovery) and low temperature. For the analysis of plants under water stress, plant tissues were harvested for gene expression analysis only when they displayed the same wilting characteristics, i.e., lack of turgor recovery, in the youngest leaves. Consequently, the time required for wilting varied among plants. We previously used this same strategy to induce drought stress in coffee plants and observed that the water potential in the leaves, even among different species, was homogeneous, resulting in reduced variation in the gene expression analysis [36]. For the low-temperature treatments, the plants were placed in a cold chamber for 12 h daily for at least 6 days. We chose water stress as a treatment because it is the field condition that most limits plant growth [37]. The low-temperature treatment was selected because *E. globulus* is adapted and cultivated in cold regions [5-7].

We also used in our assays seedlings/plants of *E. globulus* subjected to light treatment. For these experiments, the seedlings were grown in either the light or the dark. In addition, the stem apex of six-month-old plants were covered with aluminium foil and grown under greenhouse conditions. The reason to include such treatments was because we have been using light in our studies on modulation of lignin biosynthesis in *E. globulus* (Araújo and Mazzafera, unpublished data).

The qPCR technique facilitated the discrimination of genes expressed during different treatments among plants of the same or different species [38,39]. However, to validate these comparisons, the analysis must consider genes that have constitutive expression to serve as references for the analysis of plant material from different sources [14,15]. The selection of these genes is used to evaluate the stability of gene expression in different tissues, using various bioinformatics tools. In the present study, two bioinformatics tools were used that differ in terms of data analysis. The software *GeNorm* analyses the data comprehensively, establishing a reference value or M-value (stability value). The candidates with the highest values are eliminated until only two candidates remain [40]. The tool *NormFinder* only analyses variations between samples and variations within each sample group [41]. Subsequently, the variances between and within groups are used to determine the stability value, for which smaller values are considered most stable. Although *NormFinder* provides a more detailed analysis as compared with *GeNorm*, both tools are widely used for the validation of normalisers.

In the evaluations performed in the present study using *GeNorm* and *NormFinder*, few consistencies were observed among the data generated using both methodologies, as also observed in other studies with eucalyptus [27]. This was mainly observed when data were compared separately. Some few exception were observed: for example, the water stress treatment (DRT column in Table 1A) showed that for both methodologies, the best endogenous gene pair in *E. urograndis* was *H2B* /*UBQ*. However, consistency appeared when more comprehensive analyses involving different species and treatments were carried out, what then highlighted the *IDH* gene. In the analyses related to the low-temperature treatment, the genes *IDH* and *SAND* were common in the analyses using *GeNorm* and *NormFinder*. However, in addition to *IDH*, *ACT* appeared to be stable in the analyses distinguishing the tissue type (apex or base) in the samples from for the stress and low-temperature treatments.

*IDH* was also the most common gene in the *GeNorm* and *NormFinder* analyses for the material subjected to different light regimens. It is interesting to observe that the gene *UBQ* was also identified in the *E. globulus* greenhouse material using *GeNorm*, while *ACT* was identified using the *in vitro* material with *NormFinder*. The results reflect differences in the experimental conditions and the developmental stages of the plants.

*IDH*, *SAND* and *ACT* were the genes identified here as the most frequent in the gene pairs indicated in the analyses using *GeNorm* and *NormFinder*. *IDH*, *SAND* and *ACT* perform different roles related to housekeeping functions in plant cells. SAND is a member of a large family of proteins that are found in many organisms, which was identified initially in *Saccharomyces cerevisiae* and subsequently in *Schizosaccharomyces pombe, Caenorhabditis elegans*, *Drosophila melanogaster*, mouse, human, and *A. thaliana*[42]. SAND protein is involved in membrane trafficking, enabling the fusion of the vesicle to the vacuole [43]. IDH is a mitochondrial enzyme involved in the oxidative decarboxylation of isocitrate in the Krebs cycle to produce α-ketoglutarate and CO_2_, a reaction coupled with the conversion of NAD^+^ to NADH. Five isoforms of IDH are found in *A. thaliana*. Four of these enzymes are expressed in all tissues, and only one is specific to the pollen grain [44]. ACT is an important plant cytoskeleton protein that has a pivotal role in several cellular processes involved in the regulation of cell growth and morphology [45].

de Oliveira et al. [27] evaluated the microarray data from 21,432 genes and selected the 50 most stable genes expressed in the leaves of *E. grandis* and xylem from *E. grandis* and *E. globulus*. Of these 50 genes, eight were chosen, together with seven other genes that were previously tested in the literature, for studies on the stability of gene expression in the tissues of six *Eucalyptus* species (leaf and xylem of *E. grandis*, *E. dunnii*, *E. pellita*, *E. saligna* and *E. urophylla*; xylem of *E. globulus*; and flowers of *E. grandis*). Overall, these 15 genes showed adequate expression stability in the tissues analysed, but the best results were obtained with the genes *Eucons04*, *Eucons08*, and *Eucons21*, which were selected from the microarray analyses. *Eucons04* putatively encodes a protein similar to cyclin-dependent protein kinases (CDKs). *Eucons08* encodes a transcription elongation factor SII (TFIIS). *Eucons21* encodes a protein similar to aminoacyl-tRNA synthetases. All three genes showed similarities with genes from *Ricinus communis*. Among the genes used in our study, only H2B was included in the study of de Oliveira et al. [27], and it was moderately expressed. In our results, the *H2B/A-Tub* pair was consistently observed in the general analyses of drought and cold treatments in *E. uroglobulus* using the *NormFinder* software. Our data showed that the stability value places *H2B* as the fourth most stable gene, after *ACT*, *IDH*, and *SAND*. The differences observed between the data obtained in this study and that of de Oliveira et al. [27] could reflect differences in the species used and the treatments applied.

Other authors evaluated the stability of the genes used herein in eucalyptus. Almeida et al. [30] used the genes coding for 18S ribosomal RNA (18S), actin 2/7, histone H2B, NADP-isocitrate dehydrogenase, polyubiquitin, SAND protein, α-tubulin, TIP41-like protein, translation elongation factor 2, a expressed protein without determined function (33380), and a putative RNA-binding protein (EUC12) to study the formation of adventitious roots in *E. globulus*, of which the first eight genes were the same genes used in our study. de Almeida et al. [30] identified the best gene pairs as *NADP-Isocitrate Dehydrogenase/SAND* using *GeNorm* software, and *Histone H2B/α-Tubulin* using *NormFinder*. However, among the 13 genes, *UBQ* and *IDH* displayed the highest stability in two eucalyptus clones (rust-resistant and susceptible) exposed to biotic (*P. psidii*) and abiotic (acibenzolar-S-methyl) stresses [29]. In choosing the reference genes in studies on the acclimatisation of *E. globulus* to the cold, Fernández et al. [28] used the genes for elongation factor 1-a, actin, α-tubulin, protein phosphatase 1A, SAND, and ubiquitin C, concluding that the genes for ubiquitin, elongation factor 1-a and α-tubulin were the most stable.

## Conclusions

The data of Boava et al. [29], Fernández et al. [28], de Almeida et al. [30], and the current study indicate that the most frequent stable genes in the various analyses performed in different eucalyptus species and under various conditions are *ACT*, *UBQ*, *SAND*, *IDH*, and *A-Tub*, with *IDH* as the most stable. Therefore, apparently these five genes could be used as reference genes in a wide range of treatments in eucalyptus research where gene expression is evaluated. Given that the use of more than one reference gene [46] or even several genes [47] have been recommended to minimise errors in the estimation of gene expression, we propose that *IDH* and at least two more of the five genes listed could be used in a wide range of studies of biotic and abiotic stresses in eucalyptus.

## Methods

### Plant material

The low-temperature and water stress experiments were performed with *E. uroglobulus* (*E. urograndis* x *E. globulus* Labill), *E. globulus* Labill and *E. urograndis* (*E. urophylla* S.T. Blake x *E*. *grandis* Hill ex-Maiden), which were kindly provided by the Fibria Cellulose SA (São Paulo, Brazil). Upon receipt, the seedlings were maintained in a greenhouse at room temperature until the low-temperature and drought trials were performed. The plants for the low-temperature trials were grown in 10 L pots containing a mixture of soil and sand (1:1, v/v). The plants for the water stress experiments were transplanted into 10 L pots containing a soil-sand mixture (2:1, v/v) and were maintained at three plants per pot. At the time of the experiments, the plants were 90 days old in the cold experiment and 120 days old in the water stress experiment.

### Low temperature experiments

All the plants were maintained in a greenhouse at room temperature during the day. At the end of the day, half of the plants were transferred to cold and dark chambers, and the other half were transferred to chambers that were only dark and without temperature control. The plants were kept in these chambers for 12 h. The average values of the minimum and maximum temperature were, respectively, 3.2 and 5.7°C for the trial with *E. urograndis,* 6.1 and 9.1°C for *E. globulus*, and 5.7 and 8.8°C for *E. uroglobulus*. The cold experiments lasted seven (*E. urograndis*), eight (*E. globulus*), and ten (*E. uroglobulus*) days. In the control dark chamber, the average temperature ranged from 20 to 26°C. In the greenhouse, the temperature ranged from 22 to 30°C during the day.

### Water stress experiments

In these trials, 120-day-old plants grown in a greenhouse with no temperature control were divided into three groups (control, water stress, and water stress recovery). The control plants were watered daily. For the plants subjected to water stress, the irrigation was interrupted. When each individual plant showed symptoms of wilting in the younger leaves at 12:00 pm on a given day, without recovery on the morning (8:00 am) next day, the plant tissues were collected. This strategy indicated that the plant had lost the capacity to recover water turgor during the night [36]. Because the plants were only collected when showing constant signs of wilting, the development of stress in each plant occurred at different times. In general, the experiments lasted an average of 13 (*E. uroglobulus*), 19 (*E. globulus*), and 21 (*E. urograndis*) days. In the third group, the plants that did not recover their water turgor in the morning were watered, and after recovery, which occurred at approximately 16 h after irrigation, the tissues were collected.

### Light treatment

**Figure 2:**
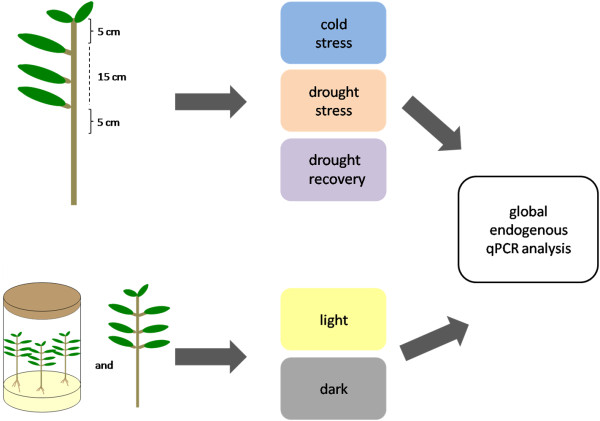
**A schematic diagram of the treatments used in the characterisation of the pattern of endogenous gene expression in eucalyptus.** Gene expression under low temperatures, drought, and recovery from drought treatments was analysed in *E. globulus*, *E. uroglobulus*, and *E. Urograndis* plants. Only *E. globulus* was used in the light and dark trial. The seedlings were cultivated *in vitro*, and the six-month plants were grown in the greenhouse.

The seedling of *E. globulus* originating from seeds germinated *in vitro* were subjected to light treatments. The seeds were sterilised with 70% alcohol for 2 min, followed by four washes with 2% hypochlorite solution and five washes with sterile water. The seeds were subsequently placed in vials containing 2.5% (w/v) phytagel, prepared with 0.5X Murashige and Skoog media (MS; [48]). The vials were placed either under light (25°C, 12 h photoperiod, 120 μmols photons.m^-2^.s^-1^) or in the dark (25°C) for 15 days (Figure 2). After this period hypocotyls were collected for analysis. To compare the results obtained with seedlings hypocotyls with plants at a more advanced stage of development, the apexes of six-month-old *E. globulus* plants were either covered with aluminium foil or uncovered for 30 days. The plants were maintained in the greenhouse without temperature control.

### Tissue collection

At the end of the water stress and low-temperature trials, the leaves were removed, and stem collection was performed. The first 5 cm from the apex (denominated apex) was collected, the following 15 cm were discarded, and the following 5 cm were collected (denominated base) (Figure 2). The apex represented the region where longitudinal cell growth is predominant, and the base represented the region where radial growth increases in intensity. The collected tissues were immediately frozen in liquid N_2_ and stored in a biofreezer (−80°C). Hypocotyls segments from seedlings grown *in vitro* were cut from the middle of the hypocotyl, frozen in liquid N_2_, and stored in a biofreezer (−80°C) (Figure 2).

In all experiments three biological replicates were used, each one composed by five plants (water and low temperature experiments) or ten seedlings (light experiment). For each replicate three technical replicates were made in the qPCR analysis.

### Nucleic acid manipulation

Total RNA extraction from the stem segments of the plants under stress was performed according to Chang et al. [49], with slight modifications. Briefly, 750 μL of extraction buffer was heated to 65°C in 2 mL Eppendorf tubes (1 tube per extracted sample), and 7.5 μL of 2-mercaptoethanol was added to each tube. Approximately 100 mg of sample was macerated in N_2_ and added to each tube followed by intense agitation in a vortex and cooling at room temperature. Subsequently, the RNA was extracted once with 750 μL of chloroform:isoamyl alcohol (24:1), mixed for 2 min, and centrifuged at 10,000 rpm for 10 min at 4°C. The supernatant was collected, and after the addition of 188 μL of a solution of 10 M LiCl, the RNA was precipitated for 12 h at 4°C. Then, the samples were centrifuged at 10,000 rpm for 30 min at room temperature. The supernatant was then discarded, and the pellet was dissolved in 200 μL of SSTE (37°C). A total of 200 μL of chloroform:isoamyl alcohol (24:1, v/v) was added. After manual agitation by tube inversion, the mixture was centrifuged at 10,000 rpm for 15 min, and the supernatant was collected. Subsequently, 1 mL of absolute ethanol was added to the supernatant and incubated at −20°C for 2 h. The samples were centrifuged at 14,000 rpm for 20 min at 4°C, and the supernatants were discarded. The pellet was washed with 75% ethanol and centrifuged at 14,000 rpm for 20 min at 4°C. The final supernatant was discarded, and the pellet was dried at room temperature. The dried RNA pellet was dissolved in 20 μL of water treated with diethylpyrocarbonate. Total RNA was extracted from the seedling hypocotyls using TRIZOL reagent (Invitrogen, Carlsbad, CA, USA). All of the extracted RNA was treated with DNAse (turbo DNA-*free*, Ambion), and the cDNA synthesis was performed using the SuperScript^R^ VILO^TM^ cDNA synthesis kit (Invitrogen). The quantification of the RNA concentration from each sample was performed in a Qubit fluorometer (Invitrogen), and the RNA quality was assessed on a 1% (m/v) agarose gel using ethidium bromide with subsequent visualisation under UV light.

### Primer design

The sequences of the genes used were obtained from four public banks related to *Eucalyptus* ( http://bioinformatics.psb.ugent.be/webtools/bogas, http://www.phytopkmdzome.net/eucalyptus.php, http://eucalyptusdb.bi.up.ac.za, and http://www.polebio.scsv.ups-tlse.fr/eucatoul/). The primers were designed using the internet software Primer3 [50] according to the following parameters: 18 and 30 bp in length, 60°C melting temperature, 40-60% GC content, and a 50–150 bp amplified fragments length.

Nine genes were selected for the stability study (Table 2): elongation factor 1-alpha (*EF1*), ß-tubulin (*B-Tub*), actin (*ACT*), ubiquitin (*UBQ*), SAND protein (*SAND*), isocitrate dehydrogenase (*IDH*), histone (*H2B*), α-tubulin (*A-Tub*), and 18S ribosomal RNA (*18S*). The choice of these genes was based on previous studies with *Eucalyptus*[28,30]. A pair of primers was designed for each gene, and for the UBQ gene, one pair of primers was designed based on the sequence of *E. globulus* and another based on the sequence of *E. urograndis* (Table 3). For each primer pair, the amplification efficiency was determined based on the slope of the standard curve for each of the genes.

**Table 2:** **The gene identification numbers and names of *Eucalyptus *genes and their orthologous genes and functions in *A. thaliana***

**Gene ID**	**Gene name**	***A. thaliana *gene**	**Function**	**Reference**
*EF1a*	Translation elongation factor	At1g18070	Translation factor activity, nucleic acid binding, GTP binding, GTPase activity;	[51]
*B-Tub*	β-Tubulin	At5g62690	GTPase activity, protein binding, structural molecule activity	[52]
*ACT*	Actin	At5g09810	Structural constituent of cytoskeleton	[53]
*UBQ*	Ubiquitin	At4g05050	Signalling complexes for protein degradation, translation control, DNA repair, endocytosis regulation, protein traffic	[54]
*SAND*	SAND protein	At2g28390	Intracellular vesicular transport, biogenesis and vacuole signalling	[17]
*IDH*	Isocitrate dehydrogenase	At1g54340	Isocitrate dehydrogenase (NADP+) activity, oxidoreductase activity, acting on the CH-OH group of donors, NAD or NADP as acceptor	[55]
*H2B*	Histone H2B	At5g22880	Structural constituent of the eukaryotic nucleosome core	[51]
*A-Tub*	α-Tubulin	At5g19780	Structural constituent of cytoskeleton, microtubule-based processes	[56]
*18S*	RNA ribosomal 18S	At3g41768	Cytosolic small ribosomal subunit, translation	[57]

**Table 3:** Gene identification and primers sequences used in the qPCR analyses

**Gene**	**Primer**	** Primer sequence (5’-3’)**	Amplicon (bp)^*^	Amplification efficiency (%)^**^
Elongation factor-1α	Forward	CCTGTCCTTGATTGTCACACTTCC	130	110
	Reverse	CCATTCCAGCATCACCGTTCTTC		
Ubiquitin (*E. gobulus*)	Forward	TCCGTCAAAAGCGAACAGA	173	97
	Reverse	CATTTCCCTCCAGATTACCC		
Ubiquitin (*E. urograndis*)	Forward	GGACTTTCGTTCGTTTTGGT	107	97
	Reverse	GTGATTTGGGGAGGGTTTG		
Actin	Forward	AGATGACCCAGATTATGTTTGAGACCTTC	122	97
	Reverse	ACCATCACCAGAATCCAACACAATACC		
SAND protein	Forward	TGGGTCACACAGGATTTTGA	130	100
	Reverse	CTCCCAGCAAAAAGATCTCG		
Isocitrate dehydrogenase-NADP	Forward	AGTTTGAGGCTGCTGGAATC	100	103
	Reverse	CTTGCATGCCCACACATAAC		
Histone H2B	Forward	AACAAGAAGCCCACCATCAC	142	96
	Reverse	ACAACTTCCTCCTCGCTCAC		
α-Tubulin	Forward	CCAGTGAACAAATGCCCTCT	92	107
	Reverse	TGATCAGCAACAACACAGCA		
Ribossomal 18S	Forward	CATGGCCGTTCTTAGTTGGT	71	95
	Reverse	TAGCAGGCTGAGGTCTCGTT		
β-Tubulin	Forward	GATGGGGACGCTATTGATTT	225	100
	Reverse	CTTGGGTTGATGAGTTTCAGG		

* Melting temperature = 60°C for all primers. **E=10^(-1/slope)-1.

### Real time quantitative RT-PCR (qPCR)

The qPCR reactions were performed using a iCycler iQ5 (Bio-Rad). The reactions contained a final volume of 10 μL and included 5 μl of QuantiFast^TM^ SYBR Green – PCR Mix (Qiagen), 0.4 μM of primers, 3 μL of cDNA, and 1.6 μL of autoclaved MilliQ water. The thermocycling conditions were 95°C for 3 min, followed by 40 cycles of 95°C for 10 sec and 60°C for 30 sec and 71 cycles of 60°C for 30 seconds. The reactions were performed in triplicate, and for each biological replicate, technical triplicates were performed. Control reactions, which contained no DNA template, were conducted for all of the experiments (NTC – Non Template Control).

### Data analysis

Determination of the best normaliser gene or best gene pair was performed using two software programmes: *NormFinder*[41] and *GeNorm*[40]. Given that *NormFinder* allows the selection of up to two genes and *GeNorm* allows the selection of more than two, we chose to work with the best combination of two genes. Expression levels were determined as the necessary number of cycles for the amplifications reach a threshold fixed in the exponential phase of PCR reaction (CT) [58]. The CTs were transformed into quantities using PCR efficiencies [40] in order to use *GeNorm* and *NormFinder*.

## Abbreviations

EF1: Elongation factor 1-alpha; B-Tub: ß-tubulin; ACT: Actin; UBQ: Ubiquitin; SAND: SAND protein; IDH: Isocitrate dehydrogenase; H2B: Histone; A-Tub: α-tubulin; 18S: 18S RNA ribosomal; qPCR: Reverse-transcription followed by quantitative real- time Polymerase Chain Reaction; RNA: Ribonucleic Acid; PCR: Polymerase Chain Reaction; cDNA: Complementary Deoxyribonucleic Acid.

## Competing interests

The authors declare no competing interest.

## Authors’ contributions

JCMSM and PA contributed equally to this work, as they performed the experiments, analysed the data and helped to write the manuscript draft. URS helped to perform RNA extraction and some of the experiments. MSB analysed the data and helped to write the manuscript draft. JOFV produced the plants and gave information about their agronomical characteristics. PM designed and supervised the study, wrote the final version of the paper. All authors read and approved the last version of the manuscript.
